# Supplementary material for: Phosphate deprivation restricts bacterial degradation of the marine polysaccharide fucoidan
Source: Nat Microbiol. 2026 Jan 22;11(2):391–405. doi: 10.1038/s41564-025-02240-z (PMC12872454; doi:10.1038/s41564-025-02240-z)
Supplement: Supplementary file 1 — Supplementary Figs. 1–4. [file 41564_2025_2240_MOESM1_ESM.pdf]

nature microbiology

Supplementary information https://doi.org/10.1038/s41564-025-02240-z

Phosphate deprivation restricts bacterial degradation of the marine polysaccharide fucoidan

In the format provided by the authors and unedited

Supplementary Figures

Phosphate deprivation restricts bacterial degradation of the marine polysaccharide fucoidan

Authors: Yi Xu[1,2,3,4,6], Bowei Gu[1,3,4,6], Huiying Yao[2,6], Mikkel Schultz-Johansen[1,3,4], Isabella Wilkie[4], Leesa Jane Klau[5], Yuerong Chen[1,3,4], Luis H. Orellana[4], Finn Lillelund Aachmann[5], Mahum Farhan[1], Greta Reintjes[1], Silvia Vidal-Melgosa[1,3,4], Dairong Qiao[2], Yi Cao[2] ✉ & Jan-Hendrik Hehemann[1,3] ✉

Affiliations:

[1]Faculty of Biology and Chemistry, University of Bremen, Bremen, Germany

[2]Microbiology and Metabolic Engineering Key Laboratory of Sichuan Province, Key Laboratory of Bio-Resource and Eco-Environment of Ministry of Education, College of Life Sciences, Sichuan University, Chengdu, Sichuan, China

[3]Center for Marine Environmental Sciences, MARUM, University of Bremen, Bremen, Germany

[4]Max Planck Institute for Marine Microbiology, Bremen, Germany

[5]Norwegian Biopolymer Laboratory (NOBIPOL), Department of Biotechnology and Food Science, NTNU Norwegian University of Science and Technology, Trondheim, Norway

[6]These authors contributed equally: Yi Xu, Bowei Gu, Huiying Yao

✉email: cyi@scu.edu.cn; jhhehemann@marum.de

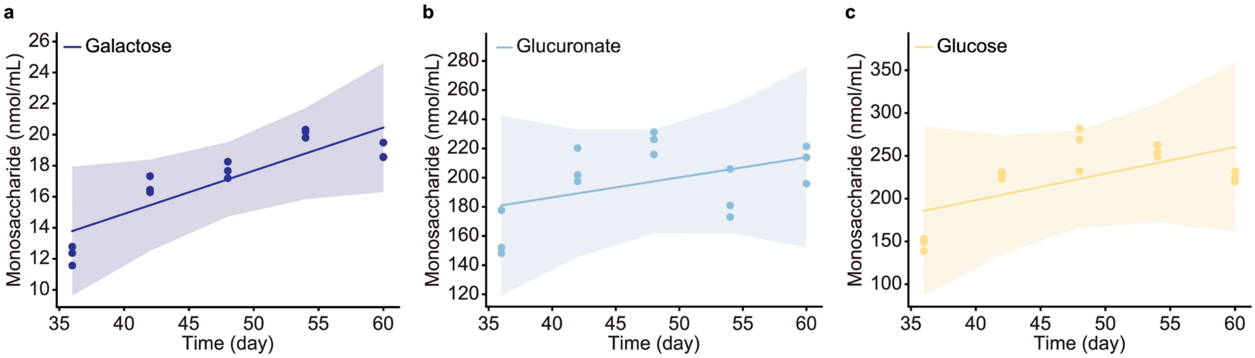

Supplementary Fig. 1: Monosaccharides that did not show significant linear changes in *Glossomastix* cultures from day 36 to day 60. a–c, Correlation analysis for galactose (r = 0.87, P = 0.05207), glucuronate (r = 0.52, P = 0.3741) and glucose (r = 0.65, P = 0.2367) in PLY432 cultures along with incubation time. The experiment was performed in independent triplicate (n = 3), the fitting was performed using the mean of triplicate. P-value was derived from two-sided Pearson correlation test. The shade indicates the 95% confidence interval around the linear regression line.

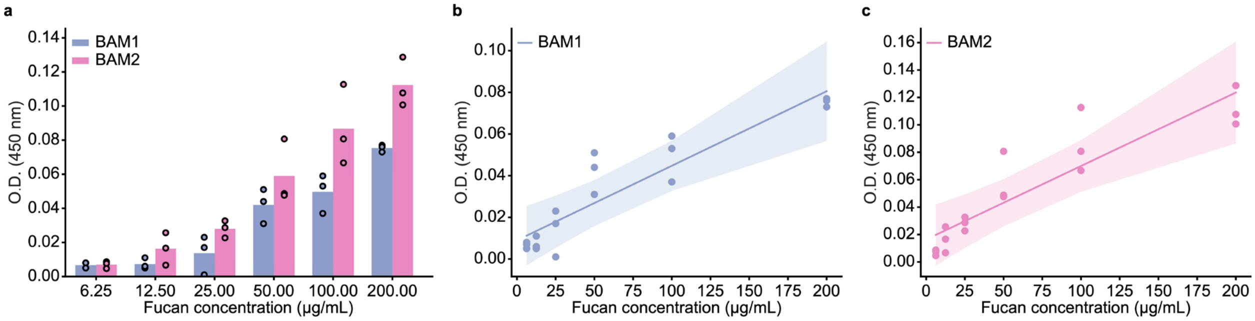

Supplementary Fig. 2: Enzyme-linked immunosorbent assay (ELISA) of *Glossomastix* fucoidan. **a,** Binding of monoclonal antibodies BAM1 and BAM2 at different concentrations of *Glossomastix* fucoidan was evaluated with ELISA and binding intensity was read at an OD of 450 nm. **b-c,** Correlation analysis for BAM1 (r = 0.95, P = 0.003274) and BAM2 (r = 0.95, P = 0.00379) with different concentration of fucoidan. The experiment was performed in triplicate (n = 3). P-value was derived from two-sided Pearson correlation test. The shade indicates the 95% confidence interval around the linear regression line.

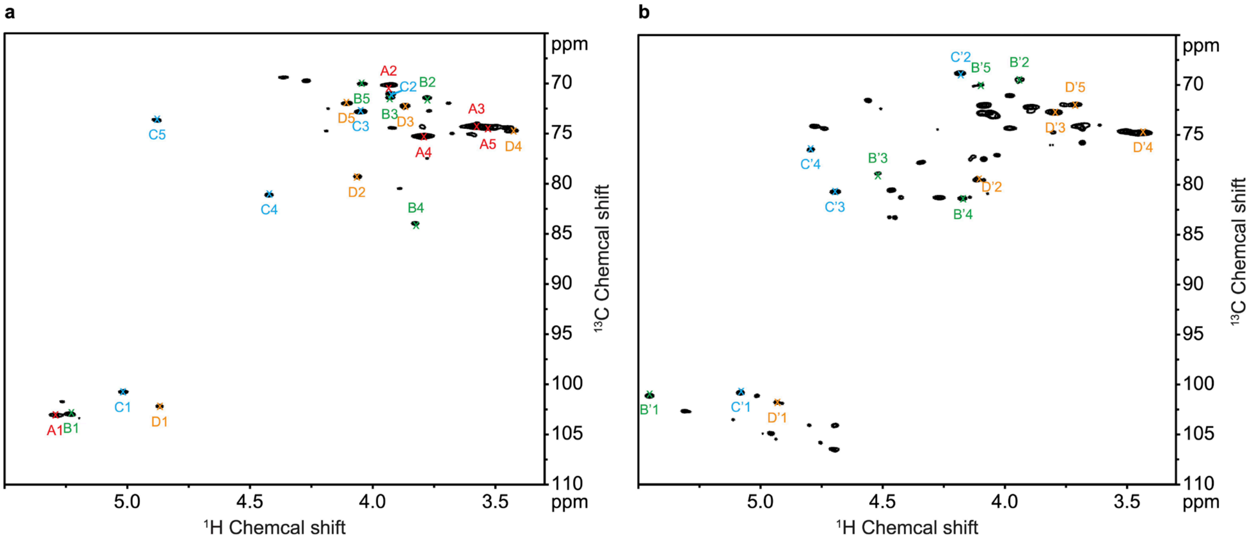

Supplementary Fig. 3: HSQC spectrum for *Glossomastix* fucoidan. a, ^{1}H-^{13}C HSQC spectrum for desulfated fucoidan from *Glossomastix*. Correlations are annotated with letter that refers to spin system and number that indicates the carbon number for position within residue. A: α-D-GlcA*p* (red); B: α-L-Fuc*p* (green); C: α-D-GalA*p* (blue) and D: β-L-Rha*p* (orange). **b,** ^{1}H-^{13}C HSQC spectrum for purified fucoidan from *Glossomastix*. Correlations are annotated with letter that refers to spin system and number that indicates the carbon number for position within residue. B': α-L-Fuc*p* (green); C': α-D-GalA*p* (blue) and D': β-L-Rha*p* (orange). The sample was dissolved in D_2O (200 µL, 99.96% D), spectrum recorded at 25°C and 800 MHz. ^{1}H chemical shift internally referenced to the residual water signal (4.75 ppm) and ^{13}C chemical shift referenced indirectly to DSS based on $^{1}H/^{13}C$ frequency ratio = 0.251449530.

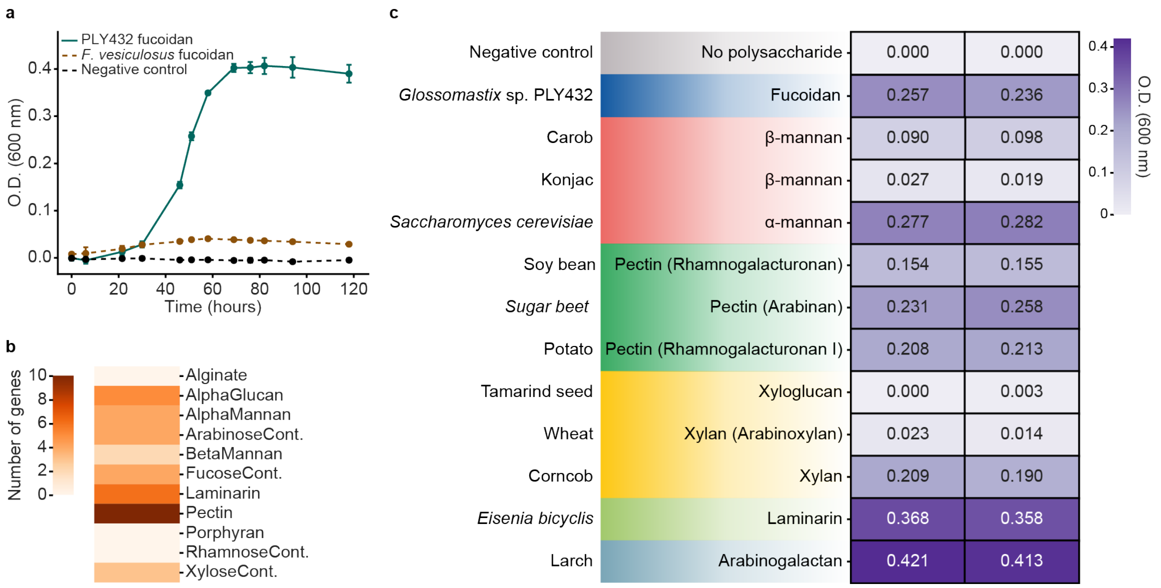

Supplementary Fig. 4: Consumption of different polysaccharides by *V_227*. a, Growth of *V*_227 in MMT-CA medium with microalgae and macroalgae fucoidan. All groups use 0.03% (w/v) Bacto^{TM} casamino acid as the phosphate source, so the medium already contains ~ 48.9 μM phosphate. The growth experiment was performed in independent triplicate (n = 3), and error bars are the standard deviation of the mean. **b,** The number of genes targeting different polysaccharides based on CAZy annotations and known substrates of annotated CAZymes. **c,** Growth of *V*_227 in MMT-KDP medium with different polysaccharides. The experiment was performed in independent two replicates (n = 2).
