# Supplementary material for: Phosphate deprivation restricts bacterial degradation of the marine polysaccharide fucoidan
Source: Nat Microbiol. 2026 Jan 22;11(2):391–405. doi: 10.1038/s41564-025-02240-z (PMC12872454; doi:10.1038/s41564-025-02240-z)
Supplement: Supplementary file 2 — Reporting Summary [file 41564_2025_2240_MOESM2_ESM.pdf]

nature portfolio

Corresponding author(s): Prof. Dr. Jan-Hendrik Hehemann

Last updated by author(s): Dec 9, 2025

Reporting Summary

Nature Portfolio wishes to improve the reproducibility of the work that we publish. This form provides structure for consistency and transparency in reporting. For further information on Nature Portfolio policies, see our Editorial Policies and the Editorial Policy Checklist.

Statistics

For all statistical analyses, confirm that the following items are present in the figure legend, table legend, main text, or Methods section.

n/a	Confirmed	
☐	☒	The exact sample size (*n*) for each experimental group/condition, given as a discrete number and unit of measurement
☐	☒	A statement on whether measurements were taken from distinct samples or whether the same sample was measured repeatedly
☐	☒	The statistical test(s) used AND whether they are one- or two-sided<br>*Only common tests should be described solely by name; describe more complex techniques in the Methods section.*
☒	☐	A description of all covariates tested
☐	☒	A description of any assumptions or corrections, such as tests of normality and adjustment for multiple comparisons
☐	☒	A full description of the statistical parameters including central tendency (e.g. means) or other basic estimates (e.g. regression coefficient) AND variation (e.g. standard deviation) or associated estimates of uncertainty (e.g. confidence intervals)
☐	☒	For null hypothesis testing, the test statistic (e.g. *F*, *t*, *r*) with confidence intervals, effect sizes, degrees of freedom and *P* value noted<br>*Give P values as exact values whenever suitable.*
☒	☐	For Bayesian analysis, information on the choice of priors and Markov chain Monte Carlo settings
☒	☐	For hierarchical and complex designs, identification of the appropriate level for tests and full reporting of outcomes
☐	☒	Estimates of effect sizes (e.g. Cohen's *d*, Pearson's *r*), indicating how they were calculated

Our web collection on statistics for biologists contains articles on many of the points above.

Software and code

Policy information about availability of computer code

Data collection	MagIC Net v3.2, TopSpin v3.5 and v4.0.1, Chromeleon v7.2, SMRT Portal software v2.3.0, ZEN2011 (Carl Zeiss, Germany), SoftMax® Pro Software 7, MARS (SPECTROstar® Nano absorbance plate reader) and PurityChrom v5.09.069.
Data analysis	MUSCLE v3.8.31, IQ-TREE v2.2.2.6, HiCanu v2.2, checkM v1.2.2, Bowtie2 v2.3.5.1, Samtools v1.7, CoverM v0.6.1, GTDB v214.1, GTDB-tk v2.3.2, dRep v3.4.3, Prokka v1.14.5, HMMER v3.3.2, dbCAN v11, Diamond blastp v2.0.14.152, SulfAtlas database v1.3, Transporter Automatic Annotation Pipeline, MetaCyc database, BlastKOALA, Prodigal v.2.6.3, Python 3.9, R v4.3.1, MPI Bioinformatics Toolkit, TVBOT, Tree of Life (iTol) and Growthcurver.

For manuscripts utilizing custom algorithms or software that are central to the research but not yet described in published literature, software must be made available to editors and reviewers. We strongly encourage code deposition in a community repository (e.g. GitHub). See the Nature Portfolio guidelines for submitting code & software for further information.

Data

Policy information about availability of data

All manuscripts must include a data availability statement. This statement should provide the following information, where applicable:

- Accession codes, unique identifiers, or web links for publicly available datasets
- A description of any restrictions on data availability
- For clinical datasets or third party data, please ensure that the statement adheres to our policy

All relevant data supporting the results of this study are available in the paper and its supplementary information. The 18S rRNA gene sequence of Glossomastix sp. PLY432 has been deposited in NCBI under accession number PP265255. The genomic data of V_227 has been deposited in NCBI under accession number PRJNA1070871. The raw data of HPAEC-PAD plus the resulting data tables used for calculation are currently being deposited on the Pangaea data repository https://www.pangaea.de/. A digital object identifier for these data will follow. Please note the tables have been also submitted as supplementary files.

Research involving human participants, their data, or biological material

Policy information about studies with human participants or human data. See also policy information about sex, gender (identity/presentation), and sexual orientation and race, ethnicity and racism.

Reporting on sex and gender	NA
Reporting on race, ethnicity, or other socially relevant groupings	NA
Population characteristics	NA
Recruitment	NA
Ethics oversight	NA

Note that full information on the approval of the study protocol must also be provided in the manuscript.

Field-specific reporting

Please select the one below that is the best fit for your research. If you are not sure, read the appropriate sections before making your selection.

☒ Life sciences ☐ Behavioural & social sciences ☐ Ecological, evolutionary & environmental sciences

For a reference copy of the document with all sections, see nature.com/documents/nr-reporting-summary-flat.pdf

Life sciences study design

All studies must disclose on these points even when the disclosure is negative.

Sample size	No sample size calculations were performed.
Data exclusions	No data was excluded from the analyses.
Replication	To ensure the reproducibility of the experiments, growth experiments for both microalga and bacteria were performed in independent biological replicates. Elemental analysis, monosaccharide composition analysis, sulfate group analysis and ELISA analysis were performed in three technical replicates for purified fucan.
Randomization	Randomization was not relevant as only in vitro experiments were performed.
Blinding	Blinding was not relevant as only in vitro experiments were performed.

Reporting for specific materials, systems and methods

We require information from authors about some types of materials, experimental systems and methods used in many studies. Here, indicate whether each material, system or method listed is relevant to your study. If you are not sure if a list item applies to your research, read the appropriate section before selecting a response.

Materials & experimental systems

n/a	Involved in the study	
☐	☒	Antibodies
☒	☐	Eukaryotic cell lines
☒	☐	Palaeontology and archaeology
☒	☐	Animals and other organisms
☒	☐	Clinical data
☒	☐	Dual use research of concern
☒	☐	Plants

Methods

n/a	Involved in the study	
☒	☐	ChIP-seq
☒	☐	Flow cytometry
☒	☐	MRI-based neuroimaging

Antibodies

Antibodies used

The following commercial antibodies were used. Product and lot numbers are specified when available.

From PlantProbes (Leeds, UK) and SeaProbes (Roscoff, France).
1. BAM1*
2. BAM2*
3. BAM3*
4. BAM4*
*PlantProbes has ceased activities since 2021, but BAM mAbs are still commercially available via SeaProbes.

From Sigma-Aldrich (MO, USA).
1. Anti-Rat IgG HR-Peroxidase secondary antibody (A9037, batch #SLCH8291)

Validation

- Fucoidan specificity of BAM mAbs demonstrated in Torode et al. (2015), PlosONE 10, e0118366.
- Validation information of Anti-Rat IgG A9037: www.sigmaaldrich.com/product/sigma/a9037

Plants

Seed stocks

NA

Novel plant genotypes

NA

Authentication

NA
