# Supplementary figures and images for: Phosphate deprivation restricts bacterial degradation of the marine polysaccharide fucoidan
Source: Nat Microbiol. 2026 Jan 22;11(2):391–405. doi: 10.1038/s41564-025-02240-z (PMC12872454; doi:10.1038/s41564-025-02240-z)

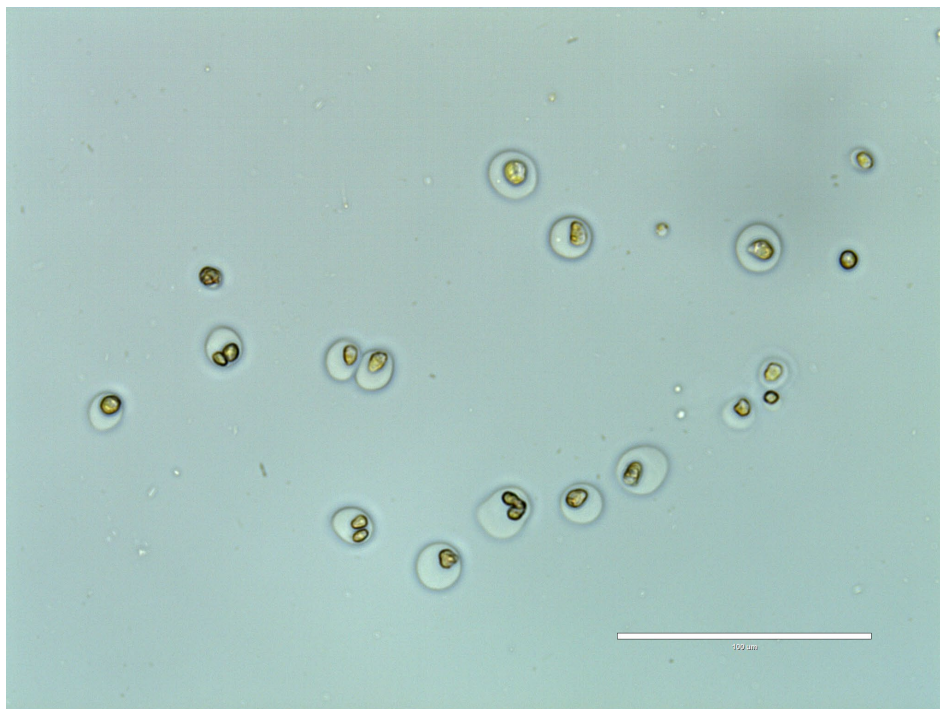

Raw picture for Extended Data Fig. 1a

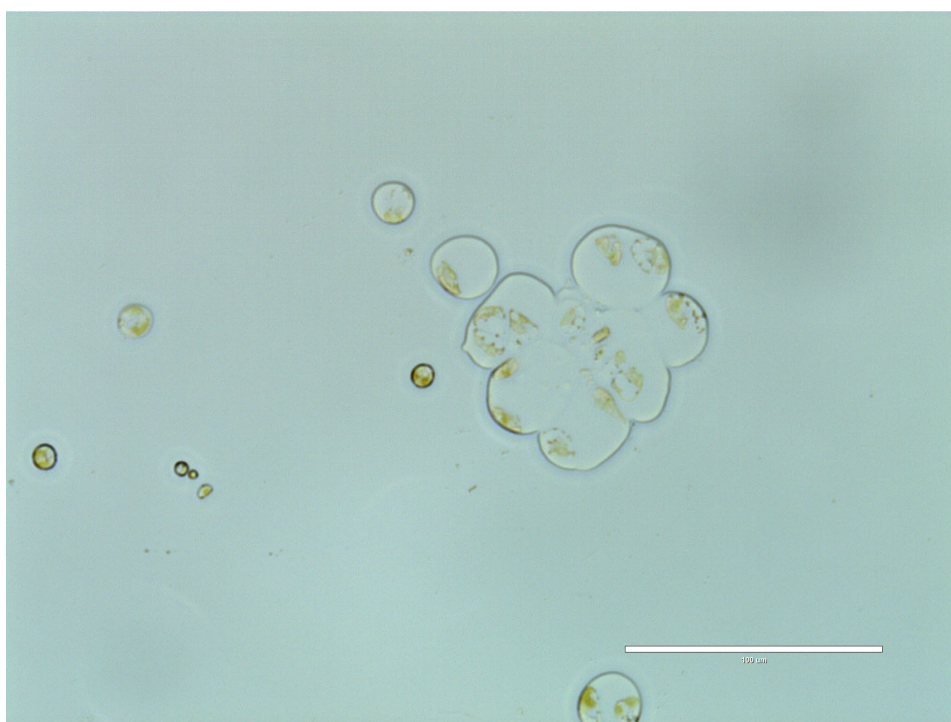

Raw picture for Extended Data Fig. 1b

Supplement: Supplementary file 11 — Unprocessed microscopic image. [file 41564_2025_2240_MOESM11_ESM.pdf]
